# How low can you go? A CBCT dose reduction study

**DOI:** 10.1002/acm2.13164

**Published:** 2021-01-15

**Authors:** Arthur J. Olch, Parham Alaei

**Affiliations:** ^1^ Radiation Oncology Program Children’s Hospital Los Angeles Los Angeles CA USA; ^2^ Department of Radiation Oncology University of Minnesota Minneapolis MN USA

**Keywords:** CBCT, image gently, imaging dose

## Abstract

**Purpose:**

Cone beam computed tomography (CBCT) is often used for patient setup based solely on bony anatomy. The goal of this work was to evaluate whether CBCT dose can be lowered to the level of kV image pair doses when used for bony anatomy‐based IGRT without compromising positioning accuracy.

**Methods:**

An anthropomorphic phantom was CT scanned in the head, head and neck, chest, and pelvis regions and setup on the linear accelerator couch with the isocenter near the planned location. Cone beam computed tomographies were performed with the standard “full dose” protocol supplied by the linac vendor. With sequentially lowering the dose, three‐dimensional (3D) matching was performed for each without shifting the couch. The standard kV image pair protocol for each site was also used to image the phantoms. For all studies, six degrees of freedom was included in the 2D or 3D matching to the extent they could be employed. Imaging doses were determined in air at isocenter following the TG‐61 formalism.

**Results:**

Cone beam computed tomography dose was reduced by 81–98% of the standard CBCT protocol to nearly that of the standard kV image pair dose for each site. Relative to the standard CBCT shift values, translational shifts were within 0.3 and 1.6 mm for all sites, for the reduced dose CBCT and kV image pair, respectively. Rotational shifts were within 0.2 degree and 0.7 degrees for all sites, for the reduced dose CBCTs and kV image pair, respectively.

**Conclusion:**

For bony anatomy‐based image guidance, CBCT dose can be reduced to a value similar to that of a kV image pair with similar or better patient positioning accuracy than kV image pair alignment. Where rotations are important to correct, CBCT will be superior to orthogonal kV imaging without significantly increased imaging dose. This is especially important for image guidance for pediatric patient treatments.

## 1 INTRODUCTION

Image‐guided radiation therapy (IGRT) is a critical component of modern radiotherapy. Most medical linear accelerators have onboard kV and cone beam computed tomography (CBCT) capabilities to facilitate IGRT. These systems along with automated couch shifts based on rigid registrations of the acquired image and the planning CT reference image provide patient positioning accuracy within about 1 mm.^1^ Most radiotherapy centers rely on IGRT for daily treatments and many perform CBCT daily or at least periodically on select patients. This daily CBCT imaging has in many cases replaced daily or weekly MV imaging performed in the era before onboard kV imaging existed. Full three‐dimensional (3D) imaging information coupled with a six degrees of freedom (6DoF) treatment couch provides state‐of‐the art patient positioning, enabling accurate treatment delivery.

Recent interest in limiting radiation exposure from diagnostic CT exams, especially for children, by tailoring the technique to the size of the patient, has carried over to considerations for IGRT dose. Although radiotherapy patients will get orders of magnitude greater dose from their treatment compared to the IGRT dose inside their treated volume, the additional daily CBCT dose outside the treated volume is comparable to the scatter dose.^2^ In addition, as the magnitude of imaging dose is inversely related to the body mass index (BMI),^3^ and children have in general lower BMI values, imaging dose magnitude is higher in children for the same imaging protocol used in adults. Therefore, there has been an interest in quantifying the dose from CBCT for IGRT and potentially reducing that dose if feasible, if only to adhere to ALARA principles. This has been stressed in the AAPM Task Group Report 180 as well, trying to reduce the dose burden from imaging while considering the risks and benefits of imaging to the patient.^4^

When imaging pediatric cases, some clinicians have elected not to use CBCT daily, but instead to perform lower dose orthogonal kV imaging daily or even weekly imaging.^5^ This lack of full 3D information may reduce the patient positioning accuracy and understanding of changes in anatomy during treatment. In this work, we have investigated the potential for orders of magnitude dose reduction for CBCT to be comparable to kV image pair dose, which is low enough to not generally cause concern. The consequence of reducing the dose for any imaging procedure is the potential for loss of information and reducing or eliminating the usefulness of the image. In this study, we focus on the scenario where only bony anatomy is being used for image registration and matching during IGRT. Where soft tissue delineation is required, CBCT dose reduction may not be possible, at least not to the extent we explored. There have been a few studies that have explored low‐dose IGRT, especially for pediatrics^6^, ^7^ but we are not aware of any prior study that aimed to answer the question, “how low can you go” in the context of CBCT dose for radiotherapy IGRT.

## 2 MATERIALS AND METHODS

An anthropomorphic phantom (Alderson Rando, Phantom Laboratory, RSD, Inc., Long Beach, CA) was CT scanned in the head, head and neck, chest, and pelvis regions and a plan for each was created in the treatment planning system so that an isocenter at each site could be created. The phantom was setup on Varian TrueBeam (Varian, Palo Alto, CA) linear accelerators with 6DoF couch with the isocenter near the planned location, but with about 0.3 to 1.5 cm translational shifts and 0.2–2.9 degree rotational shifts imposed to make the process more clinically realistic. CBCTs were performed with the standard “full dose” protocol supplied by the linac vendor. The lower dose protocols were achieved by lowering the mAs, kVp, and frame rate from standard protocols (Table 1). We started with the default kVp and mAs provided on the TrueBeam. The kVp was then reduced from 100 to 80 for head and head and neck, 125 to 100 for chest, but kept at 125 kVp for pelvis. We then progressively reduced the mAs, imaged the phantom using the new protocol, and registered the reduced dose CBCT to the planning CT. As long as the shifts agreed with the full dose CBCT within 0.3 mm and 0.3 degrees, we continued to reduce the mAs. We eventually arrived at the lowest mAs the system allowed, so to continue to reduce the dose, we lowered the frame rate. The frame rate was by default 15 frames per sec (fps) but was reduced to either 7 or 3 fps as needed to achieve lower doses. Other combinations of reducing the kVp, mAs, or frame rate are possible with potentially equivalent results but were not tested. While sequentially lowering the dose, image matching between CBCT and planning CT was performed, without applying the shifts.

**Table 1:** Image protocol parameters. NA = not applicable.

Head	kVp	mAs	Frame rate (fps)	Dose (cGy)
Standard CBCT	100	150	15	0.57
Low‐dose CBCT	80	50	15	0.11
kV pair	90/90	5	NA	0.07
Head and neck				
Standard CBCT	100	150	15	0.57
Low‐dose CBCT	80	50	7	0.05
kV pair	85/70	5	NA	0.06
Chest				
Standard CBCT	125	270	15	1.87
Low‐dose CBCT	100	90	3	0.08
kV pair	100/80	5	NA	0.08
Pelvis				
Standard CBCT	125	1080	15	6.77
Low‐dose CBCT	125	90	3	0.16
kV pair	100/75	10/5	NA	0.13

Although the auto match feature is commonly used in many clinics, manual image matching can also be performed and can produce slightly different shift results. For consistency of image matching in this study, the auto match feature was used for matching for all sites except for the head, where three radiation therapists independently performed the matching in addition to the auto shift being performed. This was done to compare the auto shift algorithm with manual matching and to demonstrate that the auto shift algorithm is reasonable to rely on for this study.

The standard kV image pair protocol for each site was also used to image the phantoms. Two‐dimensional (2D)–3D auto matching was performed for head and neck, chest and pelvis phantoms while 2D–2D auto matching and manual matching were performed for the head phantom. For all studies, 6DoF was included in the 3D matching. In the case of the 2D–2D matching using kV image pairs for the head phantom, roll was excluded from the analysis due to the lack of information in those images. In addition, a secondary set of matches was performed with only translational shifts. Imaging doses were determined in air at isocenter following the TG‐61 formalism for all protocols employed.^8^ The translational and rotational shifts were tabulated for each site for the full‐dose CBCT, low‐dose CBCT, and kV image pairs.

## 3 RESULTS

**Fig. 1:**
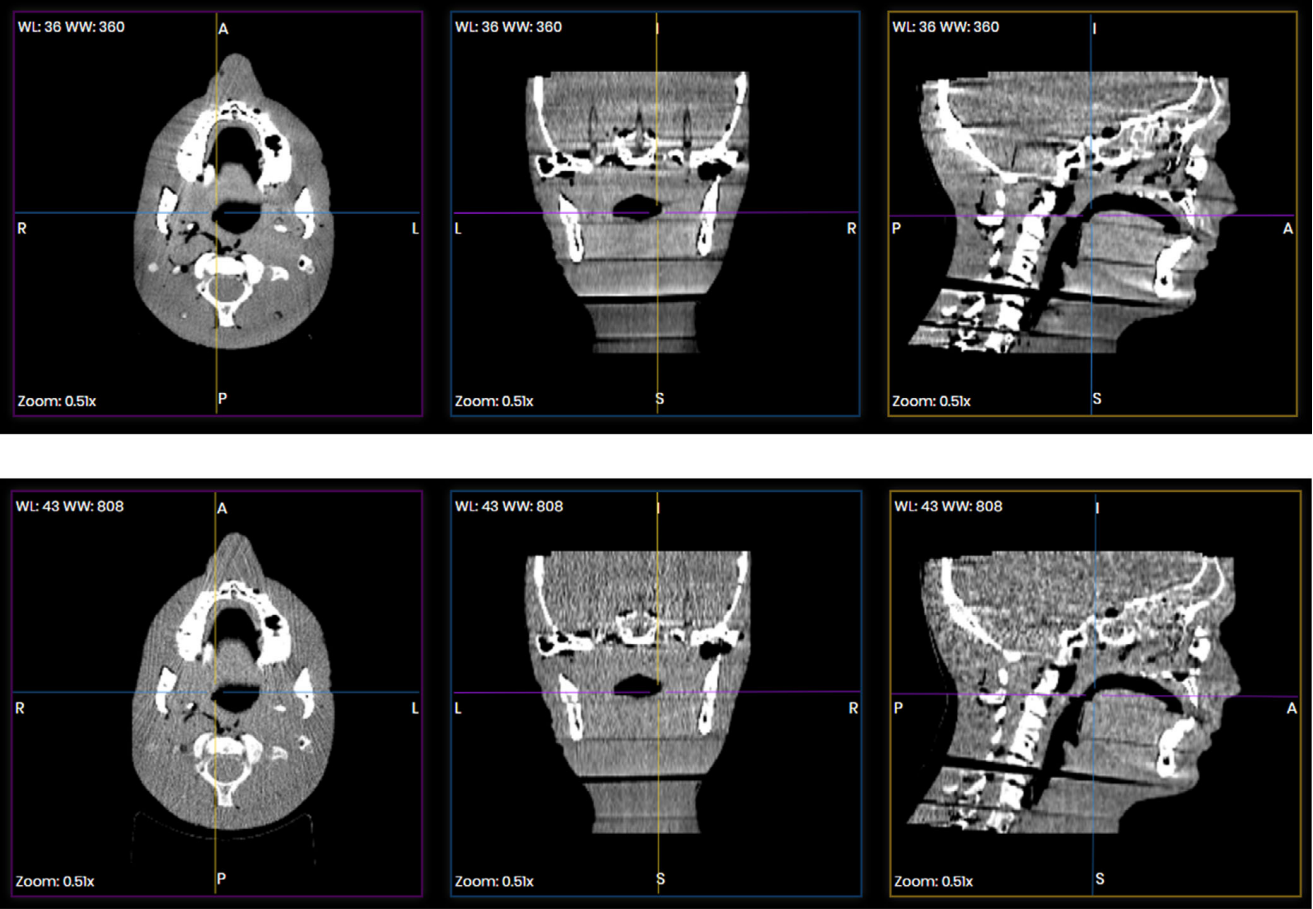
Upper panel shows full‐dose Head and Neck CBCT (100 kVp, 150 mAs, 15 fps), lower panel shows the low‐dose CBCT (80 kVp, 50 mAs, 7 fps), both registered to the planning CT.

Cone beam computed tomography dose was reduced to nearly that of the standard kV image pair dose for each site. That represented a reduction by 81‐98% of the standard CBCT protocol dose. Soft tissue image quality was of course degraded as the dose was lowered but this study only sought to retain comparable patient positioning accuracy based on bony anatomy alignment. Example images of the full and lowest dose head and neck CBCT are shown in Fig. 1. Relative to the full dose CBCT shift values, using auto shift, translational shifts for the reduced dose CBCT ranged between 0 and 0.3 mm for all sites, while shifts were slightly larger than that for the kV image pair, being 0–1.6 mm across all sites. Rotational shifts were 0–0.2 degrees for the reduced dose CBCTs for all sites while the kV image pair produced relative rotations of 0–0.7 degrees. The results are tabulated in Table 2. The CBCT deviations were repeatable to within 0.2 mm and 0.2 degrees with replicate measurements. However, the kV 2D–3D auto matched shifts were particularly variable for the pelvic site. This was especially true for roll rotations, where repeated imaging and auto matching (without applying shifts) produced values that changed by more than 1 degree. Pitch and yaw changed by several tenths of a degree for repeated tests. The shift average and standard deviations across all four sites for the CBCT images were equal to or less than that for the kV image pairs (Table 3).

**Table 2:** (a)–(e) Differences in shifts for low‐dose CBCT and kV image pair compared to standard CBCT. The actual shift from isocenter imposed prior to matching is also shown. For the head phantom, both six degrees of freedom and three degrees of freedom shifts were tested.

Shift direction	Shifts (cm/deg)	Low‐dose CBCT vs full‐dose CBCT	kV pair vs full‐dose CBCT	Rotation off kV pair vs CBCT
(a) Head auto				
Vert	0.8	−0.01	−0.15	0.07
Long	0.61	0	0.02	0
Lat	1.29	0	−0.04	0.04
Rtn	−0.7	−0.1	0.7	NA
Pitch	0.5	0.1	−0.4	NA
Roll	1.7	0	NA	NA
(b) Head manual				
Vert	0.8	0.00	0.07	0.08
Long	0.61	0.00	0.02	0.08
Lat	1.29	0.00	0.09	0.01
Rtn	−0.7	−0.03	0.43	NA
Pitch	0.5	0.10	0.07	NA
Roll	1.7	0.00	NA	NA
(c) Head and neck				
Vert	−0.76	0.02	0.02	
Long	1.22	−0.02	−0.06	
Lat	−1.02	−0.03	−0.03	
Rtn	1.6	0	0	
Pitch	−1.4	−0.2	−0.3	
Roll	−0.6	−0.1	0	
(d) Chest				
Vert	0.33	0.02	0.05	
Long	1.3	0.02	0.01	
Lat	−1.54	0.03	−0.02	
Rtn	−1.1	0	0	
Pitch	2.9	−0.2	−0.6	
Roll	0.6	0.2	0.1	
(e) Pelvis				
Vert	−1.14	0.02	0.05	
Long	1.14	0.00	0.02	
Lat	−0.82	−0.01	0.16	
Rtn	−1.9	0.00	−0.10	
Pitch	1	0.00	0.20	
Roll	−1	−0.10	^a^	

^a^ Varies between 0.1 and 1.5 degrees.

**Table 3:** Average and standard deviation of shifts (cm or degrees) compared to standard (full‐dose) CBCT based on absolute values across all sites.

	Low‐dose CBCT		kV pair	
	Ave	Std	Ave	Std
Vert	0.02	0.01	0.07	0.06
Long	0.01	0.01	0.03	0.02
Lat	0.02	0.02	0.06	0.07
Rtn	0.03	0.05	0.20	0.34
Pitch	0.13	0.10	0.38	0.17
Roll	0.10	0.08	0.05	0.07

The shifts for the head site were performed both by three experienced therapists independently, and by the auto shift function. The average translational shifts for therapists vs auto shift were in the submillimeter range for the CBCT images but differed by up to 2 mm for the kV image pairs. The average rotational shifts for the two methods differed by <0.1 degrees for the CBCTs and up to 0.47 degrees for the kV image pairs. The submillimeter difference in matching results where only translational shifts were used indicated a general equivalence of the two methods. Somewhat larger differences were found when 5DoF (roll excluded) was included, probably due to the possible interaction between translational and rotational shifts especially for manual matching.

## 4 DISCUSSION

Clinicians may be reluctant to perform daily CBCTs for fear of the added radiation dose to the patient but accept the small extra dose from daily kV orthogonal pair imaging. This mindset is particularly prevalent for pediatric treatments.^5^ In an analysis performed to estimate the risk of cancer induction for pediatric patients undergoing kV or MV IGRT, an additional 0.5 breast cancer cases per 10^4^ patient years (PY) and 0.08 lung cancers per 10^4^ PY was found for kV CBCT.^9^ Hess reviewed the peripheral dose from various types of treatments and IGRT methods and provided recommendations for imaging gently.^10^

Having full 3D volumetric imaging can be valuable in optimizing patient position, especially if a 6DoF couch is being used to achieve the corrections. It has been shown that residual shifts using CBCT after 2D image matching was about 2mm in each translational direction for the SBRT setting.^11^ For bony anatomy‐based image guidance for a wide range of anatomical sites, this study shows that it is possible to reduce the CBCT dose to a value similar to that of kV image pairs with similar or better patient positioning accuracy. Rao reported on the accuracy of low‐dose abdomen and pelvis CBCT in pediatric patients using bony landmarks and found agreement with the reference scans of 0.7 mm,^12^ however, only translational shifts were considered. In a study focused on lowering the dose for kV planar images for pediatric IGRT, a dose reduction of 20–94% was found for sites in the head and neck, thorax, abdomen, and pelvis without any apparent reduction in positioning accuracy.^6^

Where rotations are important to correct, low‐dose CBCT will be superior to an orthogonal kV image pair without significantly increased imaging dose. Due to the inherent limitations of kV image pair information, roll rotations are not well defined particularly for 2D–2D matching. The use of 2D–3D image matching in linear accelerator image guidance software is relatively new and not widely available compared to the long available 2D–2D matching method. In 2D–3D matching, the 3D CT simulation dataset is used to synthesize a range of digitally reconstructed radiograph of varying translational and rotational offsets. An optimization algorithm is used to choose the combination of 6DoF that results in the best match with the acquired 2D image pair. This can result in nondeterministic and somewhat less accurate shifts compared to those from a CBCT.^13^ Li et al described the methodology for performing 2D–3D image registration and reported on the differences in auto match results for 3D–3D vs 2D–3D images and showed that the kV image pairs resulted in >1 mm and 1 degree shift errors. These errors increased as the site location changed from head to L spine. They also found roll to be responsible for the largest deviations from the CBCT result.^13^ These image accuracy considerations are especially important for image guidance where highly precise patient positioning is required to optimize normal tissue sparing.

## 5 CONCLUSIONS

Where bony anatomy matching is appropriate, substantially lower CBCT doses are possible, equivalent to kV image pair doses, with equal or better position accuracy. These lower CBCT doses are achieved by a combination of lower kV, mA, and frame rate. This information should rationally allow for more frequent use of CBCT and substitution of CBCT for kV image pairs, resulting in improved patient position and treatment accuracy. This new approach is especially important for pediatric treatments where high accuracy is often required while at the same time imaging dose is of concern.

## AUTHOR CONTRIBUTION STATEMENT

Arthur J. Olch and Parham Alaei both contributed measurements, data analysis, and manuscript preparation and approve the final submitted version of the manuscript.
